# Late-Onset Dystrophinopathy

**DOI:** 10.7759/cureus.20531

**Published:** 2021-12-20

**Authors:** Ranjan K Singh

**Affiliations:** 1 Internal Medicine, Anti-Retroviral Therapy Centre, District Hospital, Khagaria, IND

**Keywords:** muscular dystrophy, maternal inheritance, dystrophin, duplication of exon, creatine kinase

## Abstract

Dystrophinopathy is a spectrum of muscular dystrophies resulting from absolute to relative deficiency of dystrophin - a protein essential for muscle fiber integrity. This includes a severe form called Duchenne muscular dystrophy, a mild form called Becker muscular dystrophy, and intermediate muscular dystrophy. Becker muscular dystrophy relates to late-onset and slow progression muscle dystrophy caused by deletions or duplications in the dystrophin gene. Individuals with this type of tardive slow progression have a life expectancy of 60 years. A patient in his late 40s presented this disease with duplication of exon 2 in the dystrophin gene.

## Introduction

Dystrophinopathy, an X-linked muscle disorder, includes a spectrum of muscle dystrophies of varying severity that includes Duchenne muscular dystrophy (DMD), Becker muscular dystrophy (BMD), and intermediate muscle dystrophy (IMD). The disorder results from a deficiency of dystrophin, a protein essential for the stability of muscle fibers, and is synthesized by the dystrophin gene located over the X-chromosome's short arm (p). The dystrophin gene is considered long, having 79 exons, and these exons are the portion of the gene where the genetic information is stored to make dystrophin protein. More than 7,000 different variants in DMD have been reported to date [1]. The overall incidence of BMD is one in 35,000 live births, with a prevalence of 2.4 people/100,000 population [2]. Almost all patients with DMD have cardiomyopathy, while BMD patients are at high risk for cardiomyopathy.

## Case presentation

**Figure 1:**
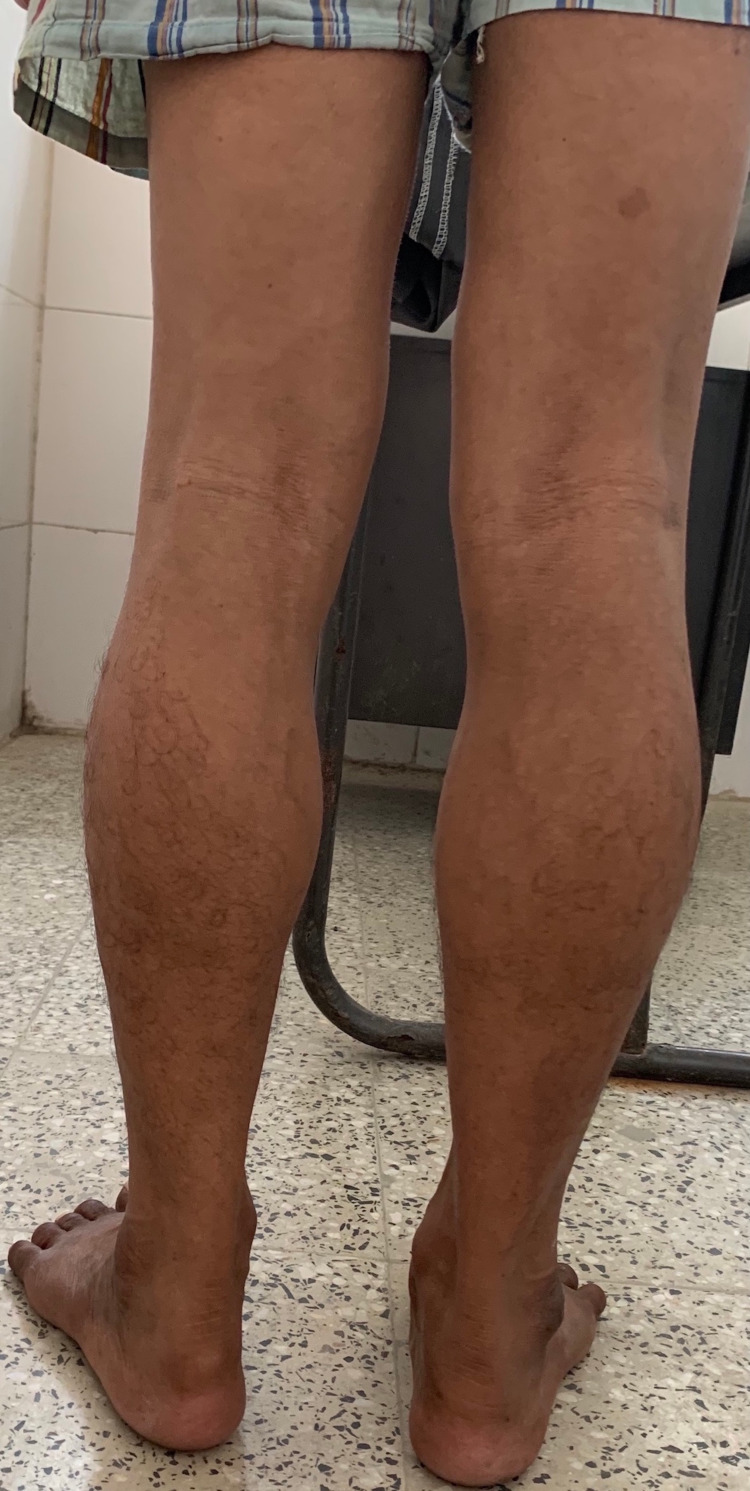
Examination of the patient showing pseudohypertrophy of calf muscles.

A man in his late 40s presented to the outpatient department with weakness in the lower limbs. He had first experienced the symptom at age 12 when he found himself falling behind his fellows while running, and it slowly became progressively worse. Although he was ambulatory, he had difficulty climbing upstairs. He had a history of maternal inheritance with his male siblings and maternal uncle, all aged 40 to 60 years, suffering from a similar pattern of muscle weakness. He had no intellectual deficit. Clinical examination revealed a waddling gait, enlargement of the calf muscles (Figure 1), and wasting of the quadriceps and gluteal muscles, giving the typical pseudohypertrophy of the calf muscles.

**Figure 2:**
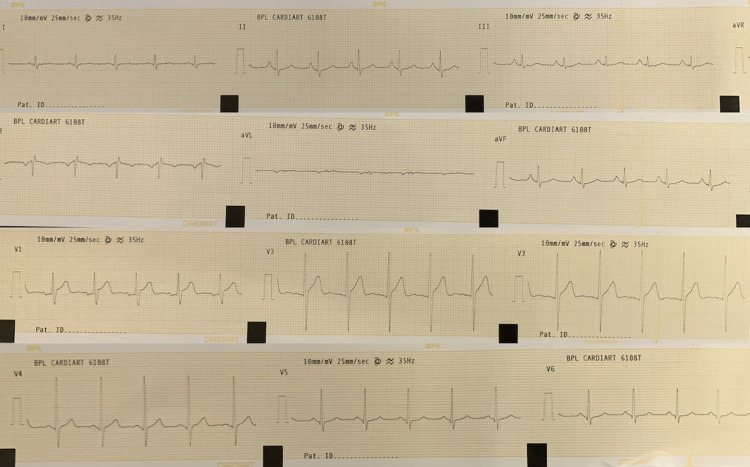
Electrocardiogram showing R/S in lead V1 > 1.

Under examination, knee jerks showed weak quadriceps contractions after tapping over knees (grade 1) and ankle jerks were absent. The patient used his arms typically when rising from the ground, showing a positive Gower’s sign. His total leukocyte count was 5.7 cells/10^9^ (reference range: 4.5-11), with neutrophils at 62% and a hemoglobin level of 11 gm/dl. Creatine kinase (units/litre) level was 1148 U/L (reference range: 22-172 U/L). A molecular genetic test (multiplex ligation-dependent probe amplification [MLPA]) showed duplication of exon 2 in the dystrophin gene. Chest X-ray and echocardiography were normal, although an electrocardiogram (ECG) showed R/S > 1 in lead V1 (Figure 2).

## Discussion

BMD is a late-onset and slowly progressive disease with pseudohypertrophy of the calf muscles (enlarged but weak calf muscles due to deposition of fat and connective tissue) and a positive Gower’s sign that results from hip muscle weakness. A creatine kinase level greater than eight times the normal range is a sign of muscle inflammation. The diagnosis is confirmed with a molecular test that revealed exon 2 duplication in the dystrophin gene. An ECG showed R/S > 1 in lead V1, but the patient had normal cardiac parameters. Besides R/S in V1, other ECG features of BMD are a deep Q wave in inferolateral leads, a short PR interval, and a long corrected QT (QTc) [2].

Two-thirds of cases have a history of maternal inheritance and the remaining show de novo point mutation of the dystrophin gene (X_p_21) [3]. A study found more than 1,000 mutations, resulting in different types of phenotypes [4]. The dystrophin gene mutations involve deleting one or more exons in 68% of cases, while duplication of single or multiple exons occurs in 11% of cases [5,6]. Duplication in the dystrophin gene occurs in exons 2-10; however, duplication of exon 2 is the most common. The outcome of deletion/duplication of exons can be in-frame mutation, i.e., the number of nucleotides in deleted/duplicated exon is divisible by three, otherwise, it is a frameshift mutation. The cell reads a gene in a group of three bases resulting in the synthesis of a shorter protein with the partial function of dystrophin. This leads to tardive slow progression of muscular dystrophy and a life expectancy of 60 years [6].

Differentials of BMD include polymyositis, limb-girdle muscular dystrophy, storage disease myopathy, and spinal muscular atrophy. They closely mimic BMD, and pseudohypertrophy and Gower’s sign distinguish these conditions. In addition to proximal muscle weakness, tongue fasciculation and atrophy of bulbar or brain stem muscles are the features of spinal muscular atrophy. In storage disease myopathy, a patient has pain and muscle weakness during physical activity. The presence of dystrophin gene mutation in a molecular genetic test confirms BMD. In this case, a multidisciplinary approach that includes frequent cardiac evaluation and physical rehabilitation is required.

## Conclusions

Pseudohypertrophy of the calf muscles and a positive Gower’s sign always indicate proximal muscle weakness in adults. A molecular test using the MLPA method is done to confirm mutations in the dystrophin gene. A cardiac evaluation should also be done at regular intervals as cases of BMD are at high risk of developing dilated cardiomyopathy.
